# Factors Influencing Superimposition Error of 3D Cephalometric Landmarks by Plane Orientation Method Using 4 Reference Points: 4 Point Superimposition Error Regression Model

**DOI:** 10.1371/journal.pone.0110665

**Published:** 2014-11-05

**Authors:** Jae Joon Hwang, Kee-Deog Kim, Hyok Park, Chang Seo Park, Ho-Gul Jeong

**Affiliations:** 1 Department of Oral and Maxillofacial Radiology, Dental Hospital of Yonsei University of College of Dentistry, Seoul, Korea; 2 Department of General Dentistry, Dental Hospital of Yonsei University of College of Dentistry, Seoul, Korea; University of Palermo, Italy

## Abstract

Superimposition has been used as a method to evaluate the changes of orthodontic or orthopedic treatment in the dental field. With the introduction of cone beam CT (CBCT), evaluating 3 dimensional changes after treatment became possible by superimposition. 4 point plane orientation is one of the simplest ways to achieve superimposition of 3 dimensional images. To find factors influencing superimposition error of cephalometric landmarks by 4 point plane orientation method and to evaluate the reproducibility of cephalometric landmarks for analyzing superimposition error, 20 patients were analyzed who had normal skeletal and occlusal relationship and took CBCT for diagnosis of temporomandibular disorder. The nasion, sella turcica, basion and midpoint between the left and the right most posterior point of the lesser wing of sphenoidal bone were used to define a three-dimensional (3D) anatomical reference co-ordinate system. Another 15 reference cephalometric points were also determined three times in the same image. Reorientation error of each landmark could be explained substantially (23%) by linear regression model, which consists of 3 factors describing position of each landmark towards reference axes and locating error. 4 point plane orientation system may produce an amount of reorientation error that may vary according to the perpendicular distance between the landmark and the x-axis; the reorientation error also increases as the locating error and shift of reference axes viewed from each landmark increases. Therefore, in order to reduce the reorientation error, accuracy of all landmarks including the reference points is important. Construction of the regression model using reference points of greater precision is required for the clinical application of this model.

## Introduction

Inevitably, error due to the position change of patient occurs during every x-ray taking despite the patient alignment protocol such as the bite material. Therefore, superimposition has been used as a method to evaluate the changes of orthodontic or orthopedic treatment.

Two-dimensional (2D) cephalometric analysis, introduced by Hofrath [1] and Broadbent [2] in 1931, has been the gold standard for clinical measurement tool in orthodontics and craniofacial surgery for the last decades. In traditional analysis, superimposition using anterior cranial base is a method to show the changes due to the growth and due to orthodontic treatment. But superimposition of anatomic structures in 2D images has limitations such as difficulty in determining landmarks and overestimating changes in the superimposed direction [3], [4].

With the introduction of cone beam CT (CBCT), evaluating changes three-dimensionally after orthodontic or orthopedic treatment became possible by superimposing images. [5]–[8] Newly introduced methods of 3D superimposition include superimposing the landmarks of the bone surface, setting up a new plane orientation and superimposing a certain selected area.

Many studies reported high reliability of identifying cephalometric landmarks with CBCT, especially on multiplanar reconstruction (MPR) images compared to 3D surface models. [9], [10] Because landmarks for superimposition should have high reproducibility, recent studies superimposed CBCT images by reorientation adopting widely used landmarks/planes as reference coordinates/planes on MPR images. [11], [12] But there have not been any studies about statistical analysis and mathematical modelling on the factors influencing superimposition error. The purposes of this study were 1) to find the factors influencing 4 point plane reorientation error, and 2) to find whether the orthodontic landmarks had sufficient reproducibility as reference landmarks and as points for analyzing superimposition error.

## Materials and Methods

### Ethics Statement

This study was approved by the IRB of Yonsei University Dental Hospital (Approval number: 13-0103(2-2013-0049)). A written or verbal informed consent was not obtained by any participants because this study was a non-interventional retrospective design and all data were analyzed anonymously. The IRB of Yonsei University Dental Hospital waived the need for individual informed consent.

### Sample

In this study, the CBCT data of 20 patients (9 males and 11 females; ranging in age from 23 to 72 years, 53.6 mean age) who visited the hospital for temporomandibular joint evaluation and took CBCT for suspected condylar pathologic bone change were selected for analysis. CBCT volumetric data (Alphard3030, Alphard Roentgen Ind., Ltd., Kyoto, Japan) were taken at 80 kV, 5 mAs with scanning time of 17 s. These images were taken using ‘P mode (154 mm×154 mm FOV)’. The voxel size was 0.30 mm. All CT images were stored using DICOM 3.0 as a medical image file format (512×512 pixel) into a Window 7-based graphics workstation (Intel Core i5 3570, 4 GByte, calibrated 21.3-inch color monitor, resolution 1563×2048 pixel, NVIDIA Quadro 2000 graphic card) and subsequently transferred toward OnDemand 3D 3Dceph application (Cybermed, CA, USA). Sagittal, axial and coronal volumetric slices as well as the 3D image reconstruction were used to determine the landmark location.

### Landmarks determination

**Figure 1:**
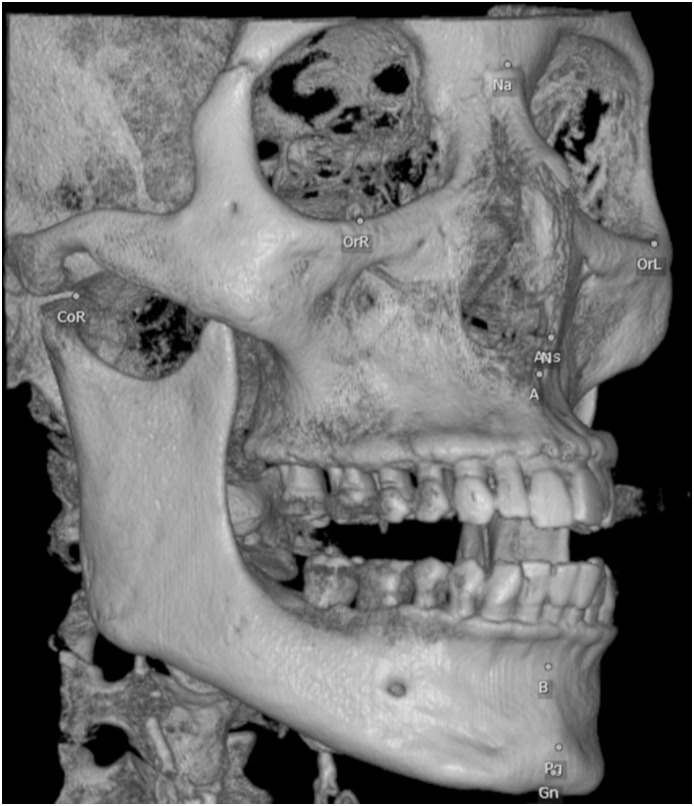
Landmarks before reorientation.

19 landmarks were located manually by ‘Tracing’ function of the software on 3D MPR images. (Figure 1).

MLWS was defined as the midpoint between the most posterior points of bilateral lesser wings of sphenoid bone. The MLSW was selected as the center of reorientation because it was close to the sella turcica in the midsagittal plane and the bilateral lesser wings had sharp posterior points, which were thought to be highly precise. All landmarks except MLWS are commonly used craniofacial structures in orthodontics and can be located without difficulty [13]. Landmarks used in the present study are defined in Table 1. Landmarks were placed by using mouse firstly on the bone surface of reconstructed CBCT images and revised secondly on MPR images. After a radiologist located all 19 landmarks, at least 1 week apart, same radiologist repeated the procedure on the same images. During the procedures, the x, y, z coordinates were gained. Locating error was obtained by measuring the absolute value of the coordinate difference and distance between the repeatedly marked landmarks.

**Table 1:** Definition of the three spatial planes of the 19 points used in this study.

PointName	Anatomicaldefinition	Sagittalview	Coronalview	Axialview
Median point ofbilateral lesserwings(MLWS)	midpointbetween themostposterior pointof bilaterallesser wing ofsphenoid bone	most PP	MP	most PP + MP
Sella turcica(S)	APP MPpituitary fossasphenoid bone	MP APP width	MP lateralwidth fossa,determinedantero-posteriorlyby theother twoslices(2)	MP APP andlateralwidth fossa
Nasion(Na)	most APfrontonasalsuture	most AP	MP	most AP+MPanterior contour
Basion(Ba)	most APforamenmagnum	most PP + LP	MP foramen,determinedantero-posteriorlyby the 2	most APforamen
AnteriorNasalspine(Ans)	most AP andmaxillaryprocess nasalfloor region	most AP	AP + MP	AP + MP
Point A(A)	most PP maxillarcurvature,betweenanteriornasalspine andsupradentalpoint	most PP	MP determinedantero-posteriorlyby the 2	AP + MP
PosteriorNasalspine(Pns)	most PP andmid-point palatinebone contour	most PP	PP + MP	PP + MP
Pogonion(Pg)	most APmandibularsymphysis	most AP	MP	AP + MP
Menton(Me)	LP mandibularsymphysis	LP	LP	LP + MP
Gnathion(Gn)	most ASPmandibularsymphysis	MA + LP	MA + LP	AP, LP + MP
Point B(B)	most PP anteriorsurfacemandibularsymphysis	most PP	MP determined antero-posteriorlyby the 2	AP + MP
Right and leftorbitale(OrR, OrL)	most AUPinfraorbitalorbital	most AP	UP + MP	Most AP
Right and leftPorion (PoR.PoL)	UP and MPexternal ridge roofauditory meatus	UP + MP	UP	MP determinedsupero –inferiorly by the 2
Right and leftCondylion(CoR, CoL)	UP point headright condyle	UP + most PP	most UP + MP	most PP
Right and leftGonion(GoR, GoL)	most PP edgebranch. Bisectiontangents posterioredge branch andlower body	most PP	most PP + MP	most PPdeterminedsupero-inferiolyby the 2

Anteroposterior point(APP), Midpoint(MP), Posterior point(PP), Lowest Point(LP), Upper point(UP), Anterior-lower Point(ALP), Anterior-upper Point(AUP), Posterior-lower Point(PLP), Highest Point(HP), Inner Point(IP).

### Reorientation procedure and Measurement of reorientation error

**Figure 2:**
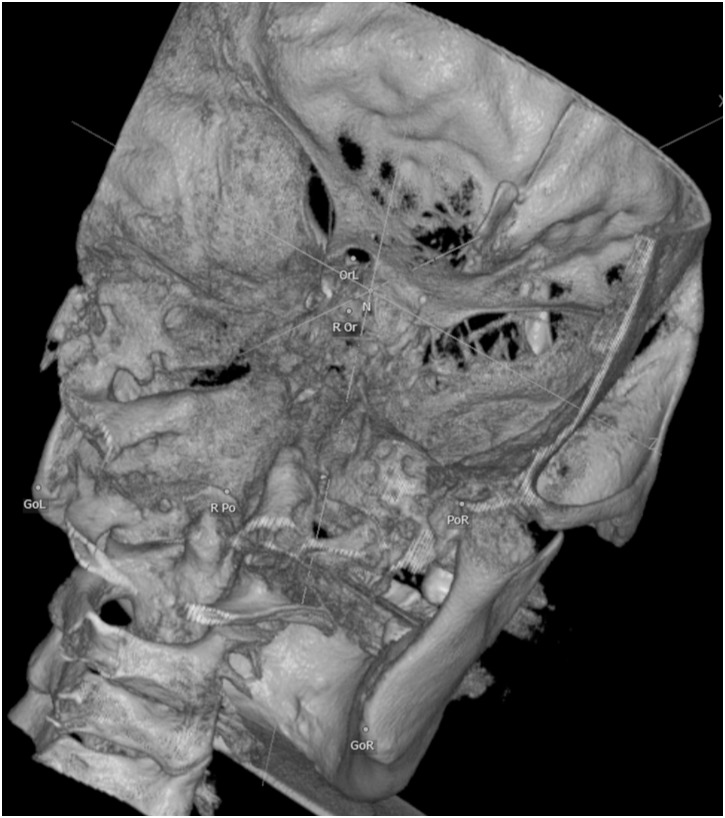
Landmarks after reorientation. N, ROr and RPo each refers to MLWS, S and Ba according to the initial setting of Ondemand 3Dceph module.

After all 19 landmarks were defined (Figure 1), the reorientation procedure was accomplished 3 times by using ‘Reorientation’ function of the software (Figure 2).

**Figure 3:**
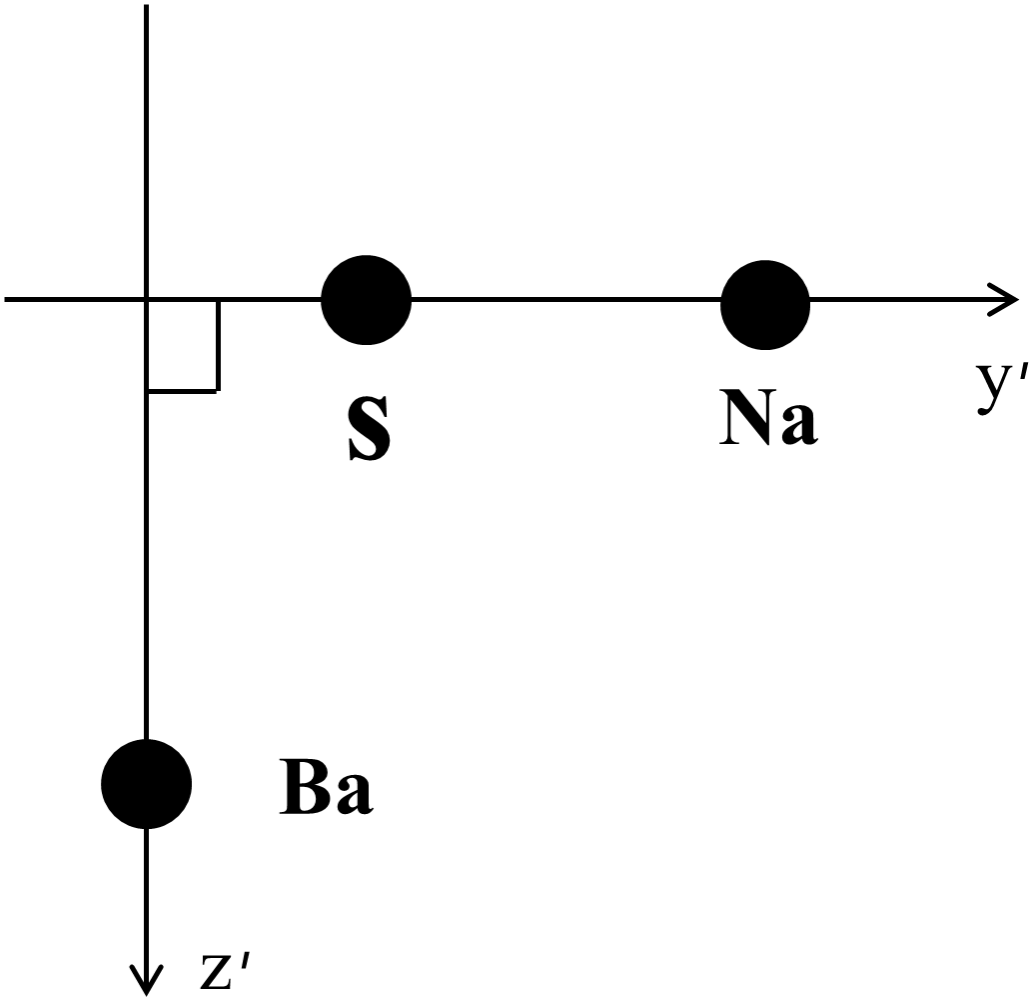
Reorientation axes. y and ź are lines parallel to y and z axis respectively.

**Figure 4:**
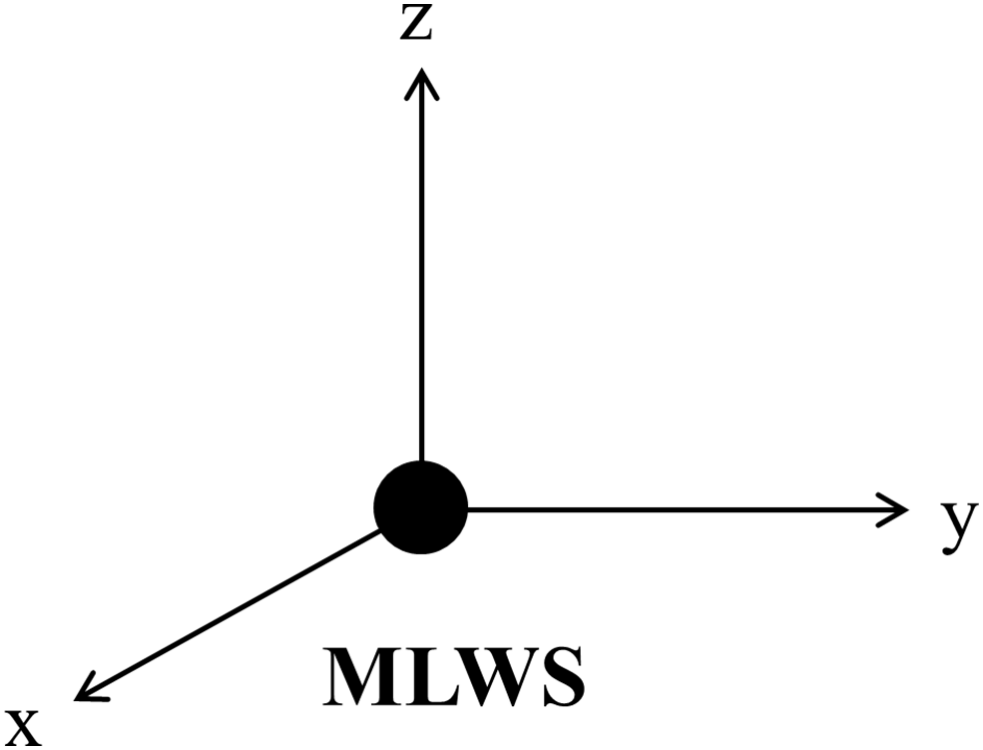
MLWS as a new starting point. x, y and z axis each represent reoriented transverse, anteroposterior and vertical axis.

Four reference landmarks out of total 19 landmarks were used to define a 3D reference co-ordinate system. Using four landmarks as the setting point is one of the simplest way of plane reorientation which can be readily applicable in the clinic. The nasion (Na), sella turcica (S) and basion (Ba) were selected for axes determination. The orientation of y (anteroposterior) axis was parallel to the line which passes through Na and S. Z (vertical) axis was parallel to the line which is orthogonal to y axis and passes through Ba. (Figure 3) Orientation of x(transverse) axis was orthogonal to the y and z axis. And MLWS was set to a new starting point of the reoriented Cartesian co-ordinate system.(Figure 4).

After plane orientation, the x, y, z coordinates were measured by the new starting point, and the vertical distance from the new x axis was measured for each point.

Reorientation error was defined as the distance of repeatedly marked landmarks after reorientation procedure.

### Statistical analysis

Statistical analysis was carried out with SPSS 20.0 (SPSS Inc., Chicago, III). Intraclass correlation Coefficient (ICC) was obtained for all coordinates of 19 landmarks. Reoriented 15 commonly used orthodontic landmarks were analyzed by stepwise linear regression tests for finding statistically significant independent variables of reorientation error.

## Results

### ICC (Intra-examiner reliability) and Locating error

**Figure 5:**
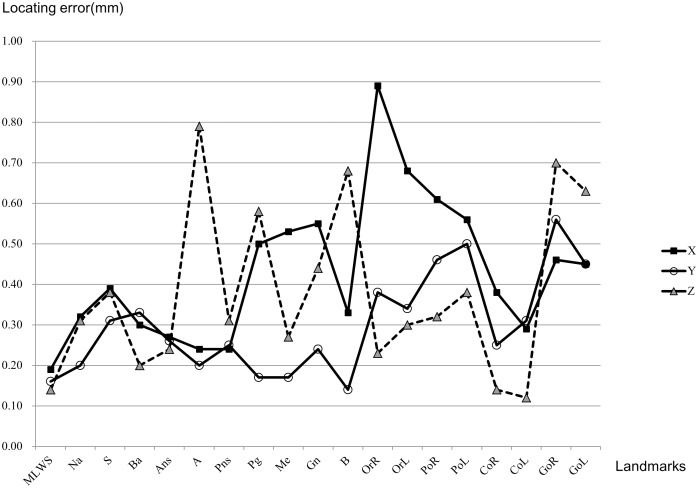
Locating error (coordinate).

ICC for x, y and z coordinates for all landmarks were above 0.99. However, there were large average locating errors in the x coordinates of OrR (0.89 mm), OrL (0.68 mm) and PoR (0.61 mm) (Table 2, Figure 5). Standard deviation was large at the locating error of A (0.74), OrR (0.58), OrL (0.48), PoR (0.45), PoL (0.55), GoR (0.52) and GoL (0.49) in comparison to MLWS (0.22), Ba (0.28), Ans (0.28), CoR (0.32) and CoL (0.29) (Table 3).

**Table 2:** Locating error (coordinate).

	Locating error		
Landmarks	X	Y	Z
MLWS	0.19(0.18)	0.16(0.17)	0.14(0.13)
Na	0.32(0.26)	0.20(0.15)	0.31(0.40)
S	0.39(0.33)	0.31(0.26)	0.38(0.33)
Ba	0.30(0.22)	0.33(0.29)	0.20(0.15)
Ans	0.27(0.22)	0.26(0.21)	0.24(0.22)
A	0.24(0.18)	0.20(0.22)	0.79(0.77)
Pns	0.24(0.19)	0.25(0.21)	0.31(0.30)
Pg	0.50(0.37)	0.17(0.13)	0.58(0.40)
Me	0.53(0.36)	0.17(0.13)	0.27(0.21)
Gn	0.55(0.40)	0.24(0.22)	0.44(0.35)
B	0.33(0.25)	0.14(0.11)	0.68(0.47)
OrR	0.89(0.60)	0.38(0.37)	0.23(0.18)
OrL	0.68(0.50)	0.34(0.29)	0.30(0.24)
PoR	0.61(0.49)	0.46(0.32)	0.32(0.25)
PoL	0.56(0.50)	0.50(0.37)	0.38(0.28)
CoR	0.38(0.35)	0.25(0.19)	0.14(0.09)
CoL	0.29(0.25)	0.31(0.27)	0.12(0.10)
GoR	0.46(0.38)	0.56(0.35)	0.70(0.47)
GoL	0.45(0.35)	0.45(0.36)	0.63(0.42)

**Table 3:** Reorientation error (RE) and factors influencing RE.

Landmarks	Reorientation error(distance)			Locating error(distance)			Distance fromreoriented X axis	Sum of angle errors from Na, S, Ba(°)
	Mean(SD)	Q1	Q3	Mean(SD)	Q1	Q3		
MLWS	0.00(0.00)	0.00	0.00	0.33(0.22)	0.18	0.38	0.00(0.00)	
Na	0.77(0.37)	0.49	0.98	0.57(0.40)	0.29	0.67	2.58(1.42)	
S	0.84(0.39)	0.55	1.06	0.73(0.38)	0.45	0.96	2.59(1.42)	
Ba	0.84(0.41)	0.54	1.11	0.56(0.28)	0.34	0.68	37.03(3.44)	
Ans	1.71(1.19)	0.90	2.17	0.51(0.28)	0.26	0.65	55.35(2.63)	1.10(0.39)
A	2.01(1.23)	1.08	2.85	0.92(0.74)	0.44	1.22	61.14(2.85)	1.04(0.37)
Pns	1.27(0.74)	0.70	1.56	0.54(0.32)	0.33	0.63	47.82(3.66)	1.60(0.57)
Pg	2.98(2.15)	1.34	3.61	0.88(0.38)	0.52	1.12	120.21(5.67)	0.66(0.22)
Me	3.08(2.16)	1.53	3.59	1.01(0.47)	0.71	1.33	126.63(6.25)	0.64(0.21)
Gn	3.01(2.33)	1.41	3.85	0.83(0.43)	0.54	1.04	124.70(6.14)	0.65(0.22)
B	2.64(1.84)	1.28	3.01	0.83(0.44)	0.47	1.09	105.81(4.73)	0.73(0.25)
OrR	1.77(1.07)	1.10	2.19	1.08(0.58)	0.70	1.49	47.01(2.93)	1.33(0.59)
OrL	1.54(0.93)	0.97	1.90	0.91(0.48)	0.56	1.10	45.90(2.77)	1.25(0.53)
PoR	1.58(1.21)	0.89	1.85	0.94(0.45)	0.64	1.17	50.14(6.09)	1.35(0.56)
PoL	1.72(1.13)	0.94	2.28	0.93(0.55)	0.61	1.13	54.43(5.41)	1.28(0.53)
CoR	1.42(0.98)	0.74	1.96	0.53(0.32)	0.34	0.65	57.98(3.06)	1.28(0.51)
CoL	1.57(1.27)	0.79	1.97	0.51(0.29)	0.31	0.68	58.80(3.74)	1.25(0.51)
GoR	2.27(1.39)	1.29	2.88	1.11(0.52)	0.80	1.45	95.37(8.04)	0.91(0.37)
GoL	2.08(1.24)	1.16	2.49	1.00(0.49)	0.62	1.26	95.14(8.12)	0.91(0.35)

### Reorientation error

Reorientation error was greater than locating error on every landmarks (Table 3). In Table 3, when locating error, vertical distance from x axis, sum of angle errors from Na, S, Ba increases, reorientation error increased as well, but all three did not show proportional change.

### Linear regression model for Reorientation error

**Figure 6:**
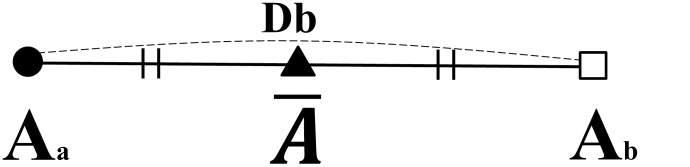
DB: Locating error (distance) of a landmark (A). 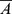
 represents an averaged landmark of A.

**Figure 7:**
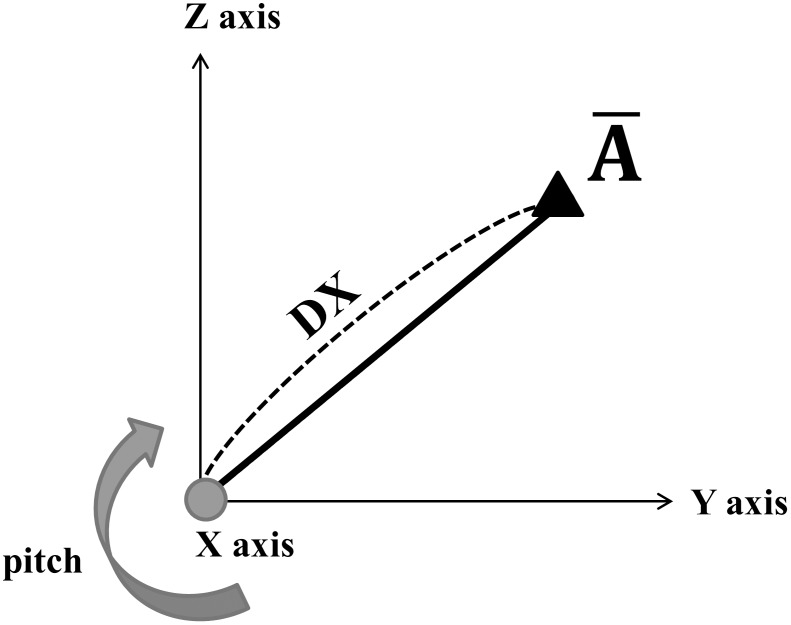
DX: Distance from X-axis to an averaged landmark. (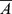) The rotated arrow around X axis represents pitch rotation

**Figure 8:**
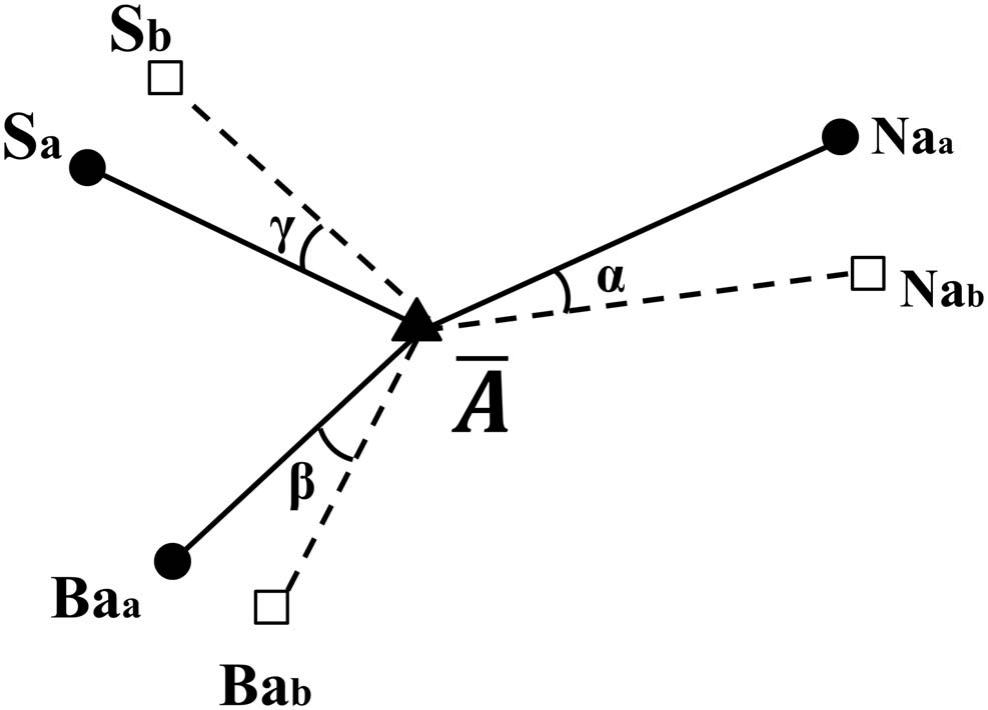
A3r: Sum of angle errors (S, Na, Ba) from an averaged landmark. (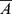) α, β and γ represent angle errors viewed from an averaged landmark (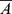) respectively.

According to the stepwise method, multiple linear regression model which explains reorientation error was found from a viewpoint of each landmark, as follows.
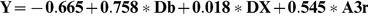
(1)


(Y =  Reorientation error,

Db =  Locating error before reorientation (Figure 6),

DX =  vertical distance from reoriented x axis (Figure 7),

A3r (α+β+γ) =  sum of angle errors of reference points located twice (Figure 8), 

All the coefficient are from multiple regression of total data).

Where a and b refer to the different measurement trial of the same image and 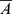 stands for the average coordinate of a landmark (A) between two trials (Figure 6, 7, 8).

The model shown in 1 was statistically significant (P = 0.000) for all T1–T2, T1–T3, T2–T3 and total analysis. The Adjusted R-square (r^2^) was around 0.23 (Table 4).

**Table 4:** Variables included in multiple regression model.

	Independent variable	B	P value	VIF	Adjusted R-square
T1–T2	Db	0.412	0.000	1.013	0.230
	DX	0.015	0.000	1.436	
	A3r	0.242	0.038	1.447	
T1–T3	Db	0.677	0.000	1.024	0.153
	DX	0.018	0.000	1.507	
	A3r	0.614	0.002	1.485	
T2–T3	Db	0.866	0.000	1.022	0.297
	DX	0.022	0.000	1.342	
	A3r	0.657	0.000	1.322	
Total	Db	0.758	0.000	1.016	0.233
	DX	0.018	0.000	1.409	
	A3r	0.545	0.000	1.397	

Outliers outside 3 standard deviations are excluded in regression analysis, VIF: variance inflation factor.

Table 4 also shows that all 3 independent variables of tested model are all statistically significant (P<0.05), and multicollinearity does not exist between the independent variables (VIF≒1).

## Discussion

### 3D reproducibility of cephalometric landmarks

Generally, marking on 2D cephalometry is quite straightforward, and 1 mm is traditionally accepted as the precise measurement. Identifying the cephalometric landmarks in 3D CBCT was reported reliable in many studies, especially on MPR images. [9], [13] But locating error is larger in 3D than 2D, and this seems to be the reason why 3D cephalometry is not widely used clinically [14].

Table 2 and 3 showed reproducibility of some widely used cephalometric points were not very high in 3D MPR images. The locating errors were about 1 mm on average. However, some points at the third quartile had 1.2–1.5 mm locating errors, and some of the locating errors were greater than 2 mm. This results correlates partially with the study of Zamora et al. [10] which reporting high errors in A, OrR, OrL, PoR, B and GoR. In addition, Hassan et al. [9] reported large locating errors (average 1.2∼2.1 mm) in PoR, PoL and GoL. In Table 2 and figure 5, most of the landmarks showed the largest locating errors on the x coordinates. These results can be assumed to have been produced due to defining of the cephalometric landmarks on lateral view without the consideration of the transversal plane.

Na, S, Ba used as reference landmarks generally showed good reproducibility.(Table 2 and 3) However, the S point tended to exhibit slightly higher locating error than Na and Ba. MLWS showed better reproducibility than Na, S, Ba in this study. The locating error of MLWS (mean 0.33, SD 0.22) was similar to the smallest locating error (mean 0.31, SD 0.19) of the upper left incisor in the study of Hassan et al. [9].

### Plane orientation system for superimposition

Superimposition methods that use 3D anatomical landmarks or surface has recently become the focus of interest. [15], [16] But when superimposition is made on bone surface landmarks or certain areas, the superimposition error increases as it gets further from the superimposition area or growth area.

Lagravère et al. [11] used 4 landmarks at the skull base, a stable structure, for carrying out plane orientation but a different superimposition method was used compared to this study. In this study, 4 landmarks were also used for plane orientation, but unlike the study above in which the new starting point was fixed on the new axes, the two were separated, making it a more flexible reorientation system.

If multiple landmarks are marked on the skull base, the results would be more accurate, but time consuming and clinically inefficient, making the reorientation system more complicated and harder to analyze statistically. So in this study, Na, S, Ba were used which have previously been used [17]–[19] or recommended [10] cephalometric landmarks in several studies, as reference points of axes set up. Also, the most accurate MLWS was used as the center of reorientation and made a plane reorientation system considering both efficiency and accuracy.

### Reorientation error

#### Increased error after reorientation

After reorientating the landmarks which were positioned repeatedly at the same image in this study, reorientation error increased than locating error (Table 3). This is because every point, including the reference landmarks, differs whenever it is located, so the landmark at each trial is reoriented by different axes and origin.

#### Factors influencing reorientation error

In a previous study [11], factors influencing reorientation were mentioned as voxel size and locating error of the center point. This study showed voxel size imposes smallest measurement uncertainty (0.25 mm) that makes errors when determining reference points and planes. And those errors can produce up to 1 mm error after transformation in one axis of reference points. Ondemand 3D program could enlarge MPR images and measure up to second decimal number and therefore, was hardly influenced by voxel size.

Same study mentioned that as the errors which are imposed at origin increase, errors of reference points increase after transformation, but, in this study there was no further quantitative explanation except the increase is not directly proportional.

Until now, the factors that produce errors during reorientation using cephalometric landmarks have never been analyzed statistically. In this study, the plane orientation error was analyzed by multiple regression. When making this model, the understanding of transformation was changed by analyzing axes change from a peripheral viewpoint (each landmark), rather than center perspective (origin and reference axes).

This linear regression model (1) could explain reorientation error (distance of a landmark after reorientation, Y) of this plane orientation system about 1/4 with 3 simple factors below.

First, Db stands for locating error of each landmark itself. As locating error before transformation (Db) increases 1 mm, reorientation error increases approximately 0.76 mm in this system. This explains that the locating error is reflected on the reorientation error up to approximately 76%.

Second, DX stands for vertical distance from reoriented x axis to each landmark. As DX increases 10 cm, reorientation error increases approximately 1.8 mm in this system. This means that mandibular chin area which is about 13 cm away from the newly set x axis can get approximately 2.3 mm reorientation error only due to its far location from reoriented x axis.

Third, A3r stands for shift of reference axes viewed from each landmark during two landmark determination trial. As one (1) degree of A3r from a point increases, reorientation error increases about 0.55 mm. This result shows that small axes shift can affect the reorientation error substantially.

This model shows that A3r with DX may have possibility to be ‘position scalar’ in a system which consists of limited number of points (eg. cephalometric analysis). A3r alone has the difficulty in explaining reorientation error. However, by analyzing A3r with DX, the approximate reorientation error can be predicted which is the outcome of vector transformation. This idea of ‘position scalar’ can be established because the locating error of reference landmarks is small enough to produce the unique value of A3r combine with DX.

Among the above values, if the accuracy of the landmarks including the reference could be improved, the Db & A3r value and the coefficient of DX would decrease, thereby reducing the reorientation error. However, in terms of the A3r value, assuming that Na, S, Ba are sufficiently accurate, the patient’s anatomical structures are likely to have a greater impact than the locating error of those three points.

The hypothesis that the distance of each point from MLWS as well as the perpendicular distance from the reoriented y, z axes could have an effect on the amount of reorientation error has been denied through regression analysis.

### Limitation of this study and works to be done

The selected Na, S, Ba points for the determination of the reorientation axes are positioned on the mid sagittal plane. Therefore, even though the location error of these points were relatively small, the pitch direction of the reoriented x axis will be the most greatly affected. This is the reason for the increase in reorientation error with increase in DX. In this model, the points with large DX can already be expected to have a large reorientation error; hence, accurate superimposition will be difficult to achieve. Thus, to promote clinical application, accurate setting of the reference landmarks will be required to reduce the value of the constant in front of DX, and further research will be needed to minimize the increase in reorientation error caused by DX.

As shown by the above data, a locating error of 1 mm on average may result in the increase of 0.76 mm in reorientation error. When the other two factors that affect the reorientation error are considered, further research necessitates the selection of accurate points with locating errors less than 1 mm.

The difference in adjusted R-square value between T1–T2, T1–T3 and T2–T3 can be explained by the low reproducibility of some of the landmarks that would have acted as outliers to weaken the explanation power of the regression model (table 4). This is another reason why this model can be considered limited yet for clinical application.

Future plane orientation exercises should utilize precise reference points rather than the well-known orthodontic landmarks. The highly precise reference points of this experiment include sharp points such as ANS and PNS, midpoints of two sharp points such as MLWS, as well as an end point of a protruding eminence such as Ba. Previous studies [11], [20] indicate that center points of foramina are also highly precise points of reference. In further studies, regression models based on such precise reference points should be evaluated for its accuracy after CBCT image superimposition based on 4 point plane orientation.

## Conclusions

In present study, 3D reproducibility of some widely used cephalometric points was not adequate for accurate evaluation of 4 point plane reorientation error. This model showed that locating error, vertical distance from reoriented x axis and shift of reference plane viewed from each landmark are important factors that explain the reorientation error.

## Supporting Information

Table S1 **Coordinates before and after reorientation (Sample Number 1).**
(XLSX)

Table S2 **Coordinates before and after reorientation (Sample Number 2).**
(XLSX)

Table S3 **Coordinates before and after reorientation (Sample Number 3).**
(XLSX)

Table S4 **Coordinates before and after reorientation (Sample Number 4).**
(XLSX)

Table S5 **Coordinates before and after reorientation (Sample Number 5).**
(XLSX)

Table S6 **Coordinates before and after reorientation (Sample Number 6).**
(XLSX)

Table S7 **Coordinates before and after reorientation (Sample Number 7).**
(XLSX)

Table S8 **Coordinates before and after reorientation (Sample Number 8).**
(XLSX)

Table S9 **Coordinates before and after reorientation (Sample Number 9).**
(XLSX)

Table S10 **Coordinates before and after reorientation (Sample Number 10).**
(XLSX)

Table S11 **Coordinates before and after reorientation (Sample Number 11).**
(XLSX)

Table S12 **Coordinates before and after reorientation (Sample Number 12).**
(XLSX)

Table S13 **Coordinates before and after reorientation (Sample Number 13).**
(XLSX)

Table S14 **Coordinates before and after reorientation (Sample Number 14).**
(XLSX)

Table S15 **Coordinates before and after reorientation (Sample Number 15).**
(XLSX)

Table S16 **Coordinates before and after reorientation (Sample Number 16).**
(XLSX)

Table S17 **Coordinates before and after reorientation (Sample Number 17).**
(XLSX)

Table S18 **Coordinates before and after reorientation (Sample Number 18).**
(XLSX)

Table S19 **Coordinates before and after reorientation (Sample Number 19).**
(XLSX)

Table S20 **Coordinates before and after reorientation (Sample Number 20).**
(XLSX)

## References

[1] Hofrath H (1931) Importance of teleroentgenograms for diagnosis of maxillary abnormalities. J Orofac Orthop 1: 232–258.

[2] Broadbent BH (1931) A new X-ray technique and its application to orthodontia. Angle Orthod 1: 45–66.

[3] Gu Y, McNamara Jr JA (2008) Cephalometric superimpositions: A comparison of anatomical and metallic implant methods. Angle Orthod 78: 967–976. 18947269 10.2319/070107-301.1

[4] Arat ZM, Rübendüz M, Akgül AA (2003) The displacement of craniofacial reference landmarks during puberty: A comparison of three superimposition methods. Angle Orthod 73: 374–380. 12940557 10.1043/0003-3219(2003)073<0374:TDOCRL>2.0.CO;2

[5] Berkowitz S (1999) A multicenter retrospective 3D study of serial complete unilateral cleft lip and palate and complete bilateral cleft lip and palate casts to evaluate treatment: Part 1 - The participating institutions and research aims. Cleft Palate Craniofac J 36: 413–424. 10499403 10.1597/1545-1569_1999_036_0413_amrsos_2.3.co_2

[6] Seckel NG, Van der Tweel I, Elema GA, Specken TFJM (1995) Landmark positioning on maxilla of cleft lip and palate infant - A reality? Cleft Palate Craniofac J 32: 434–441. 7578209 10.1597/1545-1569_1995_032_0434_lpomoc_2.3.co_2

[7] Sachdeva R (2001) SureSmile technology in a patient-centered orthodontic practice. J Clin Orthod 35: 245–253. 11345571

[8] Ashmore J, Kurland B, King G, Wheeler TT, Ghafari J, et al (2002) A 3-dimensional analysis of molar movement during headgear treatment. Am J Orthod Dentofacial Orthop 121: 18–29. 11786867 10.1067/mod.2002.120687

[9] Hassan B, Nijkamp P, Verheij H, Tairie J, Vink C, et al (2013) Precision of identifying cephalometric landmarks with cone beam computed tomography in vivo. Eur J Orthod 35: 38–44. 21447781 10.1093/ejo/cjr050

[10] Zamora N, Llamas JM, Cibrian R, Gandia JL, Paredes V (2012) A study on the reproducibility of cephalometric landmarks when undertaking a three-dimensional (3D) cephalometric analysis. Med Oral Patol Oral Cir Bucal 17: e678–688. 22322503 10.4317/medoral.17721 PMC3476034

[11] Lagravere MO, Major PW, Carey J (2010) Sensitivity analysis for plane orientation in three-dimensional cephalometric analysis based on superimposition of serial cone beam computed tomography images. Dentomaxillofac Radiol 39: 400–408. 20841457 10.1259/dmfr/17319459 PMC3520184

[12] Swennen G, Schutyser F, Barth E, De Groeve P, De Mey A (2006) A new method of 3-D cephalometry part I: The anatomic cartesian 3-D reference system. J Craniofac Surg 17: 314–325. 16633181 10.1097/00001665-200603000-00019

[13] Zamora N, Llamas J, Cibrián R, Gandia J, Paredes V (2012) A study on the reproducibility of cephalometric landmarks when undertaking a three-dimensional (3D) cephalometric analysis. Med Oral Patol Oral Cir Bucal 17: e678–e688. 22322503 10.4317/medoral.17721 PMC3476034

[14] Lagravere MO, Low C, Flores-Mir C, Chung R, Carey JP, et al (2010) Intraexaminer and interexaminer reliabilities of landmark identification on digitized lateral cephalograms and formatted 3-dimensional cone-beam computerized tomography images. Am J Orthod Dentofacial Orthop 137: 598–604. 20451778 10.1016/j.ajodo.2008.07.018

[15] Hoffmann J, Westendorff C, Leitner C, Bartz D, Reinert S (2005) Validation of 3D-laser surface registration for image-guided cranio-maxillofacial surgery. J Craniofac Surg 33: 13–18. 10.1016/j.jcms.2004.10.001 15694144

[16] Kang SH, Kim MK, Kim JH, Park HK, Park W (2012) Marker-free registration for the accurate integration of CT images and the subject’s anatomy during navigation surgery of the maxillary sinus. Dentomaxillofac Radiol 41: 679–685. 22499127 10.1259/dmfr/21358271 PMC3528203

[17] Maeda M, Katsumata A, Ariji Y, Muramatsu A, Yoshida K, et al (2006) 3D–CT evaluation of facial asymmetry in patients with maxillofacial deformities. Oral Surg Oral Med Oral Pathol Oral Radiol Endod 102: 382–390. 16920547 10.1016/j.tripleo.2005.10.057

[18] Kitaura H, Yonetsu K, Kitamori H, Kobayashi K, Nakamura T (2000) Standardization of 3-D CT measurements for length and angles by matrix transformation in the 3-D coordinate system. Cleft Palate Craniofac J 37: 349–356. 10912713 10.1597/1545-1569_2000_037_0349_sodcmf_2.3.co_2

[19] Katsumata A, Fujishita M, Maeda M, Ariji Y, Ariji E, et al (2005) 3D–CT evaluation of facial asymmetry. Oral Surg Oral Med Oral Pathol Oral Radiol Endod 99: 212–220. 15660095 10.1016/j.tripleo.2004.06.072

[20] Schlicher W, Nielsen I, Huang JC, Maki K, Hatcher DC, et al (2012) Consistency and precision of landmark identification in three-dimensional cone beam computed tomography scans. Eur J Orthod 34: 263–275. 21385857 10.1093/ejo/cjq144

